# SICE: an improved missing data imputation technique

**DOI:** 10.1186/s40537-020-00313-w

**Published:** 2020-06-12

**Authors:** Shahidul Islam Khan, Abu Sayed Md Latiful Hoque

**Affiliations:** 1 Department of CSE, Bangladesh University of Engineering and Technology, Dhaka, Bangladesh; 2 Department of CSE, International Islamic University Chittagong, Chittagong, Bangladesh

**Keywords:** Missing Data Imputation, Single Imputation, Multiple Imputation, MICE, Data Analytics

## Abstract

In data analytics, missing data is a factor that degrades performance. Incorrect imputation of missing values could lead to a wrong prediction. In this era of big data, when a massive volume of data is generated in every second, and utilization of these data is a major concern to the stakeholders, efficiently handling missing values becomes more important. In this paper, we have proposed a new technique for missing data imputation, which is a hybrid approach of single and multiple imputation techniques. We have proposed an extension of popular *Multivariate Imputation by Chained Equation (MICE)* algorithm in two variations to impute categorical and numeric data. We have also implemented twelve existing algorithms to impute binary, ordinal, and numeric missing values. We have collected sixty-five thousand real health records from different hospitals and diagnostic centers of Bangladesh, maintaining the privacy of data. We have also collected three public datasets from the UCI Machine Learning Repository, ETH Zurich, and Kaggle. We have compared the performance of our proposed algorithms with existing algorithms using these datasets. Experimental results show that our proposed algorithm achieves 20% higher F-measure for binary data imputation and 11% less error for numeric data imputations than its competitors with similar execution time.

## Introduction

In the past few years, the generation of digital data has been increased swiftly, along with the rapid development of computational power. These enable the way to extract novel insights from massive datasets, known as big data. In different disciplines such as healthcare, banking, e-commerce, and finance, data analysts are working to discover hidden knowledge from a vast volume of data [1, 2]. Quality of data is a significant concern to them for fruitful data analytics. Although the outcome of data analysis tasks depends on several factors such as attribute selection, algorithm selection, sampling techniques, etc., a key dependency relays upon efficient handling of missing values [3, 4].

Different machine learning and data mining algorithms are widely used to predict outcomes from large datasets. These algorithms usually make proper prediction unless the data used for training the algorithms are flawed. An essential step of the data analysis and mining process is the refinement of the data on which the system will be trained. This part of the data mining process is called data preprocessing, which is recognized as the most challenging part by the data analysts [5, 6]. In many cases, data is either missing or incorrectly entered by a human, which results in wrong predictions. One of the main issues regarding the quality of data is missing values. Missing values in a dataset may significantly increase computational cost, skew the outcome, and frustrate researchers [7].

Like traditional data analysis tasks, missing data is also a critical problem in big data analytics. Quality data is one of the major requirements in big data processing. However, data quality is degraded due to the presence of missing values [1, 4]. Big data usually contain various types of measurement errors, outliers, and missing values. These issues add an additional complication to data preprocessing and analysis tasks. In big data analytics, it is an important task to extract low dimensional structure from high dimensional data. Many traditional statistical procedures for missing data imputation are not well suited in the noisy and high dimensional setting of big data. Different machine learning techniques (e.g., neural network, support vector machine, etc.) cannot be applied if a dataset contains missing values [8].

It is a simple solution to ignore the observation with missing values. Usually, no significant problem occurs when there are very few observations with missing values. However, deleting a large number of observations with missing values causes a significant loss of information [9]. It also decreases the statistical power and efficiency of the data [10]. Hence reliable imputation techniques are necessary to solve the missing data issue. Imputation of missing data can help to maintain the completeness in a dataset, which is very important in small scale data mining projects as well as big data analytics.

There are some widely used statistical approaches to deal with missing values of a dataset, such as replace by attribute mean, median, or mode. Many researchers also proposed various other solutions targeting the imputation of binary, nominal, or numeric data. In this paper, we have presented a new technique for missing data imputation named Single Center Imputation from Multiple Chained Equation(SICE) which is a hybrid approach of single and multiple imputation methods. We have proposed an extension of popular *Multivariate Imputation by Chained Equation (MICE)* algorithm. We have also implemented twelve existing algorithms to impute binary, ordinal, and numeric missing values of four different datasets. We have compared the performance of our proposed algorithm with existing algorithms and found that our proposed algorithm achieves higher accuracy, F-measure, and less error than its competitors.

The rest of the paper is organized as follows. At first, we have reviewed the existing literature, which is pivotal to our research in "Background and related works" section. Then in "Proposed algorithm" section, we have presented our proposed method SICE for the imputation of different types of missing data. We have implemented some popular methods and compared the results of SICE with them using local and open-source datasets, which are discussed in "Experimental design" and "Results" sections . In "Discussions and limitation" section, we have discussed the results and limitations of our proposed algorithm briefly. Finally, the Conclusion section presents a summary of the paper.

## Background and related works

In this section, we have presented the necessary background and literature related to missing data imputation. First, we have described briefly the types of missing data. Then we have presented the literature review in two categories: single imputation and multiple imputation.

Typically missing data can be of three types:

- Missing Completely at Random (MCAR): Data are missing independently of both observed and unobserved data. For example, in a student survey, if we get 5% responses missing randomly, it is MCAR.
- Missing at Random (MAR): Given the observed data, data are missing independently of unobserved data. For example, if we get 10% responses missing for the male students’ survey and 5% missing for the female students’ survey, then it is MAR.
- Missing Not at Random (MNAR): Missing observations are related to values of unobserved data itself. For example, if lower the CGPA of a student, the higher the missing rate of survey response, then it is MNAR.

### Single imputation

Single imputation techniques generate a specific value for a missing real value in a dataset. This technique requires less computational cost. There are many types of single imputation methods proposed by the researchers. The general procedure is to pick the highest possible response by analyzing other responses. The value may be obtained by mean, median, mode of the available values of that variable. Other approaches, such as machine learning-based techniques, may also be used for single imputation. An illustrative example of how single imputation works is presented below.

In Table 1, we can see that there are two missing values in the “Income” column for serial number 2, and 5 which are represented by NA. We can run mean imputation to impute the missing values. Here, for each missing value, only one value will be imputed by the algorithm. Now we will calculate the mean of the available values of the “Income” column.

$$\begin{aligned} \hbox {Mean}= (100+100+300+200+200)/5= 180 \end{aligned}$$

**Table 1:** A dataset with missing values

Serial	Gender	Income
1	Female	100
2	Female	NA
3	Male	100
4	Female	300
5	Male	NA
6	Male	200
7	Female	200

At this point, the missing values of serial 2 and 5 will be replaced by the mean value of this column, which is 180. Table 2 represents the situation after the imputations of missing values. If there are a lot of missing data in a column, and these data are replaced by the same value, the statistical result like standard deviation, variance goes down. In single imputation, imputed values are considered as actual values. Single imputation ignores the fact that the actual value cannot be predicted for sure by any imputation method. Single imputation based methods do not consider the uncertainty of the imputed values. Instead, they recognize the imputed values as actual values in subsequent analysis. However, these values may have standard errors. These causes bias in the result [11, 12].

**Table 2:** Imputing missing values using single imputation method

Serial	Gender	Income
1	Female	100
2	Female	180
3	Male	100
4	Female	300
5	Male	180
6	Male	200
7	Female	200

In Table 3, we can see, there are some missing values in the dataset. If we use a single imputation strategy, we may take “Mode” (most frequent value) of our target column “Death Reason” to fill these missing values. In this example, the mode is “Cancer,” so all the missing data will be replaced by “Cancer.” However, if we consider the age column, then we can see that the missing values are for the *senior* patients who are more likely to die in Covid-19. So, if we just fill all the missing values using only single imputation, it may not correctly address the uncertainty of the dataset and likely to produce bias imputation.

**Table 3:** Analysis of bias for single imputation method

Serial	Age	Death reason
1	60	Covid-19
2	64	NA
3	42	Heart attack
4	67	Covid-19
5	80	NA
6	32	Cancer
7	35	Cancer
8	45	Cancer
9	88	NA
10	33	Heart attack

The followings are some prominent research of single imputation based missing data imputation techniques. Grzymala-Busse and Grzymala-Busse [13] presented a review of existing missing data handling methods in the handbook *Handling Missing Attribute Values*. They have categorized existing methods into sequential imputation and parallel imputation methods and discussed the popular sequential imputations, e.g., case deletion, assigning the most common value, concept-restricted assignment of values. A few parallel imputation methods were also discussed in their paper, e.g., rule induction, lower and upper approximation, attribute value pairing.

In [14], the authors stated the influences and risks of missing data imputation on medical data and how they impact the classification accuracy. The authors compared three averaging methods of data imputations: global average, cluster average, and class average. The importance of using classification techniques after imputation with an algorithm is also discussed in the paper.

Rahman [15] presented an imputation technique for missing healthcare data based on rule based machine learning approach. Here, the author used an algorithm, namely the Fuzzy Unordered Rule Induction Algorithm(FURIA). FURIA is an advancement of a learner algorithm called RIPPER [16]. FURIA produces a few if-then rules depending on the dataset. Later these if-then rules can be used to impute the missing values. The author compared the performance of FURIA with kNN, J48, SVM, and Mean imputation, to impute missing data and found FURIA to be better in terms of sensitivity. Accuracy of FURIA was not always promising than its competitors.

Schmitt P., Mandel J., and Guedj M. selected six of the most popular methods for missing data imputation from Google search engine and compared the methods using few open-access datasets, i.e., iris, e.coli, and breast cancer [17]. They evaluated the effectiveness of these methods using root mean square error (RMSE), Unsupervised Clustering Error, and Supervised Clustering Error. The authors found that Bayesian Principal Component Analysis(bPCA) and Fuzzy K-Means(FKM) outperform the other methods.

Amiri and Jensen [18] presented a missing data imputation technique using Fuzzy-Rough Methods. The paper helps its readers to grasp the concepts of fuzzy-rough sets along with different versions of fuzzy inference and their implementation. The paper used “KEEL,” an open-source software, as well as a library that can be used to perform advanced data-mining techniques over a dataset [19]. KEEL has the implementation of algorithms like Fuzzy-Rough Nearest Neighbor (FRNN), which is a classification algorithm. The authors considered FRNN and proposed three missing value imputation methods- Fuzzy-Rough Nearest Neighbors Imputation(FRNNI), Vaguely Quantified Rough Sets(VQRS), and Ordered Weighted Average Based Rough Sets(OWABRS). At the end, FRNNI was found to be performing best among the three proposed algorithms.

In [20], the authors compared seven imputation methods for numeric data. The algorithms are mean imputation, median imputation, predictive mean matching, kNN, Bayesian Linear Regression (norm), non-Bayesian Linear Regression (norm.nob), and random sample. They used five numeric datasets from the UCI machine learning repository and found that kNN imputation outperformed all other methods.

Support Vector Machine (SVM) is a popular classification algorithm that is widely used for missing data imputation [21, 22]. For a labeled training sample, SVM tries to find an optimal separating hyperplane such that the distance from the hyperplane to the nearest data points is maximized [23]. The larger this distance (i.e., “margin”), the lower the generalization error of the classifier. The classifier is referred to as the maximum margin classifier. The data points that are nearest to the hyperplane are called the support vectors. Several kernel functions have been introduced in SVM to reduce the computational cost for classification such as the Linear kernel, Laplacian kernel, and Polynomial kernel.

### Multiple imputation

Multiple imputation methods produce multiple values for the imputation of a single missing value using different simulation models. These methods introduce the variability of imputed data to find a range of plausible responses. Multiple imputation methods are complex in nature, but they do not suffer from bias values like single imputation. MICE algorithm, proposed by V. S. Buuren and K. Groothuis-Oudshoorn, is widely used for multiple imputation [24]. The working principle of multiple imputation techniques is illustrated next with an example.

**Fig. 1:**
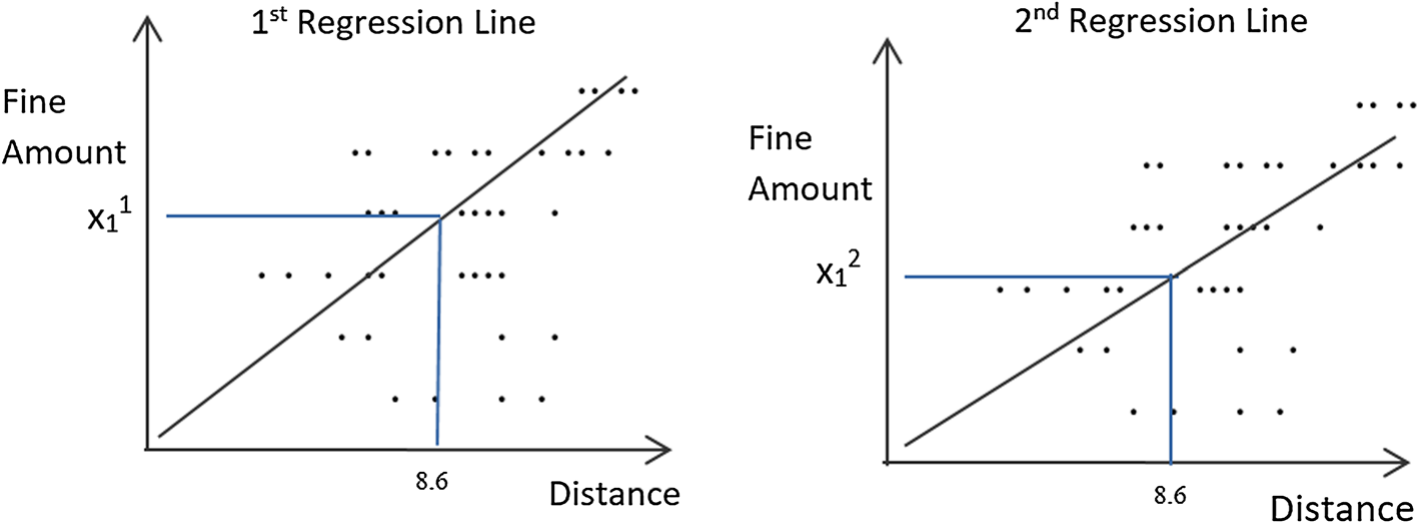
Regression lines from two sets of random 100 data taken from 1000 library fine data

In multiple imputation, each missing data are replaced with *m* values obtained from *m* iterations (where *m* > 1 and *m* normally lies between 3 to 10). Let us have a dataset of 1000 peoples (shown in Table 4) about their distance from a particular library and the amount of late fine the library has imposed on them. The dataset has some missing values in the *fine amount* column. We want to impute the missing values using multiple imputation techniques where the value of *m* is 10. In each iteration, we will run regression between “Distance from library” and “Fine Amount” by taking 100 random values. In the first imputation, we get $x_{i}^{1}$ for missing values (replacement of the ith missing value of target variable x with first regression). Similarly, in the second imputation, we take another 100 random values and run regression between “Distance from library” and “Fine Amount.” Then we fill the ith missing value with $x_{i}^{2}$ (replacement of ith missing value of target variable x with second regression). We will perform these steps ten times to get ten imputations for all missing values of the target variable. Figure 1 is an illustration of two imputations using two regression lines. Table 5 represents the results of 3 imputations.

**Table 4:** Example of 1000 library fine data with missing values

Serial	Distance from library	Fine amount
1	1.7 mi	$11
2	2.1 mi	$10
3	8.6 mi	NA
4	0.2 mi	$3
5	6.1 mi	NA
......	......	......
......	.....	.....
......	.....	.....
1000	5.3 mi	$10

**Table 5:** Multiple imputation for table 4

Serial	Distance from library	Fine amount [1st Imputation]	Fine amount [2nd Imputation]	Fine amount [3rd Imputation]
1	1.7 mi	$11	$11	$11
2	2.1 mi	$10	$10	$10
3	8.6 mi	*$17*	*$16*	*$18*
4	0.2 mi	$3	$3	$3
5	6.1 mi	*$15*	*$15*	*$16*
......	......	......	......	......
......	.....	.....	.....	.....
......	.....	.....	.....	.....
1000	5.3 mi	$10	$10	$10

**Fig. 2:**
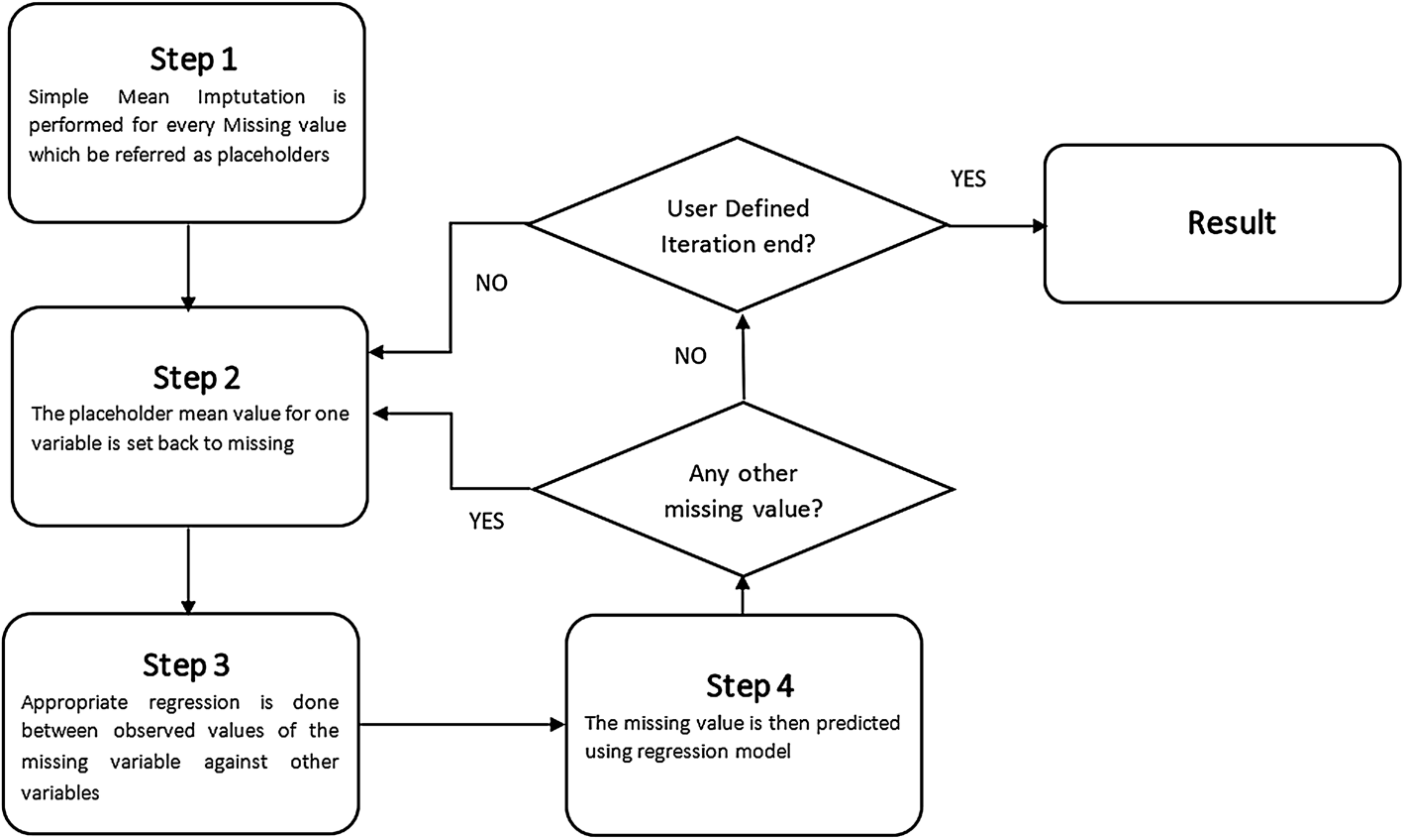
MICE flowchart

Multivariate Imputation by Chained Equation (MICE) package in “R” is the implementation of the popular MICE algorithm. MICE assumes that data are missing at random (MAR). It pretends the probability of a missing variable depends on the observed data. MICE provides multiple values in the place of one missing value by creating a series of regression (or other suitable) models, depending on its ‘method’ parameter. In MICE, each missing variable is treated as a dependent variable, and other data in the record are treated as an independent variable. The process is presented in Fig. 2.

At first, MICE predict missing data using the existing data of other variables. Then it replaces missing values using the predicted values and creates a dataset called *imputed dataset*. By iteration, it creates multiple imputed datasets. Each dataset is then analyzed using standard statistical analysis techniques, and multiple analysis results are provided. As popular single imputation methods, e.g., mean, class-mean, are likely to produce a biased imputation, multiple imputation methods could provide better results.

In the MICE package of R, there are more than twenty methods that can be set for the imputation of missing data [24]. Some methods can be applied only to binary data, and some others work for numeric data. Few methods can be used for all attribute types. Selected methods from the MICE package are discussed below.

#### Predictive mean matching

Predictive Mean Matching (PMM) is a general-purpose method for missing data imputation [25]. One advantage of PMM is that imputations are confined to the observed values. PMM can preserve non-linear relations also when the structural part of the imputation model is incorrect. Let, k is a variable with some missing values, and variable l, with no missing data, is used to impute k. The algorithm works in the following way:

1. For non-missing data, linear regression of k on l is done, which produces b (a set of coefficients).
2. A random draw from the posterior predictive distribution of b is made, which produces a new set of coefficients b*.
3. By using b*, predicted values for k are generated for all cases.
4. For the cases with missing k, a set of cases are identified that contained observed k whose predicted values are close to the predicted value with missing data.
5. From those close cases, a value is chosen randomly to replace the missing value.
6. Steps 2 to 5 are repeated for every completed dataset.

#### Logistic regression

Logistic Regression (LOGREG) [26], a popular statistical tool used to analyze a dataset for an outcome where there are one or more independent variables. In logistic regression, the dependent variable is binary. Examples of such data could be YES or NO. Logistic regression generates the coefficients to predict a logit transformation of the probability of presence of the characteristic of output:

logit(y)= $b_0+b_1X_1+b_2X_2+b_3X_3+.......+b_kX_k$ where y is the probability of the presence of the characteristic of output.

#### Polytomous logistic regression

Polytomous Logistic Regression (POLYREG) [27] method defines how multinomial target variable Q depends on a set of independent variables, $P_1, P_2, ... P_m$. This is also a generalized linear model where the random component assumes that the distribution of the dependent variable is Polynominal $(n,\pi ),$ where $\pi$ is a vector with probabilities of “success” for each category.

#### Linear discriminant analysis

Linear Discriminant Analysis(LDA) [28] calculate posterior probabilities for all incomplete cases and pick imputations, subsequently, from their posteriors. Steps for linear discriminant analysis is given below

1. Calculate the d-dimensional mean vectors from dataset for different classes
2. Calculate scatter matrices
3. Compute eigenvectors ($e_1,e_2,...,e_d$) and their associated eigenvalues ($\lambda _1$,$\lambda _2$,...,$\lambda _d$) for the scatter matrices
4. Sort eigenvectors according to the decreasing eigenvalues and choose k eigenvectors with the highest eigenvalues to form a matrix W with d $\times$ k dimension
5. Use W to transform the samples onto new subspace. This can be summarized by the matrix multiplication: Y = X $\times$ W

#### Classification and regression tree

Classification and Regression Tree (CART) [29] first examines all explanatory variables and determine which binary division of a single explanatory variable best reduces deviance in the response variable. CART and other decision tree-based algorithms have the following key elements:

- Rules to split data at a node based on the value of one variable
- Stopping rules to decide the terminal branch with no more split
- A prediction in each leaf node for the target variable

#### Bayesian linear regression

Bayesian Linear Regression(BLR) [30] is a popular statistical method. It is an approach to linear regression, where statistical analysis was undertaken within the context of Bayesian inference. Here linear regression is formed with the help of probability distributions instead of point estimates. Y, the response, is not assessed as a single value, but y is assumed to be drawn from a probability distribution. BLR aims to find out the posterior distribution for the model parameters rather than finding a single best value.

#### Amelia

Amelia is a multiple imputation method which is not included in the MICE package and a separate R package is available for it. To impute missing values for a specific dataset, Amelia uses a bootstrapping and expectation-maximization algorithm. It creates multiple imputations by multiple iterations [31]. This is helpful since later imputations can be compared to discover trends or to find better results.

### Summary

In this section, we have reviewed many research works, broadly categorized as single imputation and multiple imputation based techniques. Single imputation based approaches are computationally efficient but may significantly suffer from bias as they do not consider the uncertainty of the missing data. On the contrary, multiple imputation based approaches avoid bias and add uncertainty at the cost of high computational cost. In this era of big data, where a massive volume of data is the typical case for practical datasets, multiple imputation based approaches are challenging to implement. Considering the limitations of both single and multiple imputation based approaches, we are proposing an approach that combines the goodness of both the approaches: simplicity and uncertainty. Our proposed imputation technique is presented in the next section.

## Proposed algorithm

**Fig. 3:**
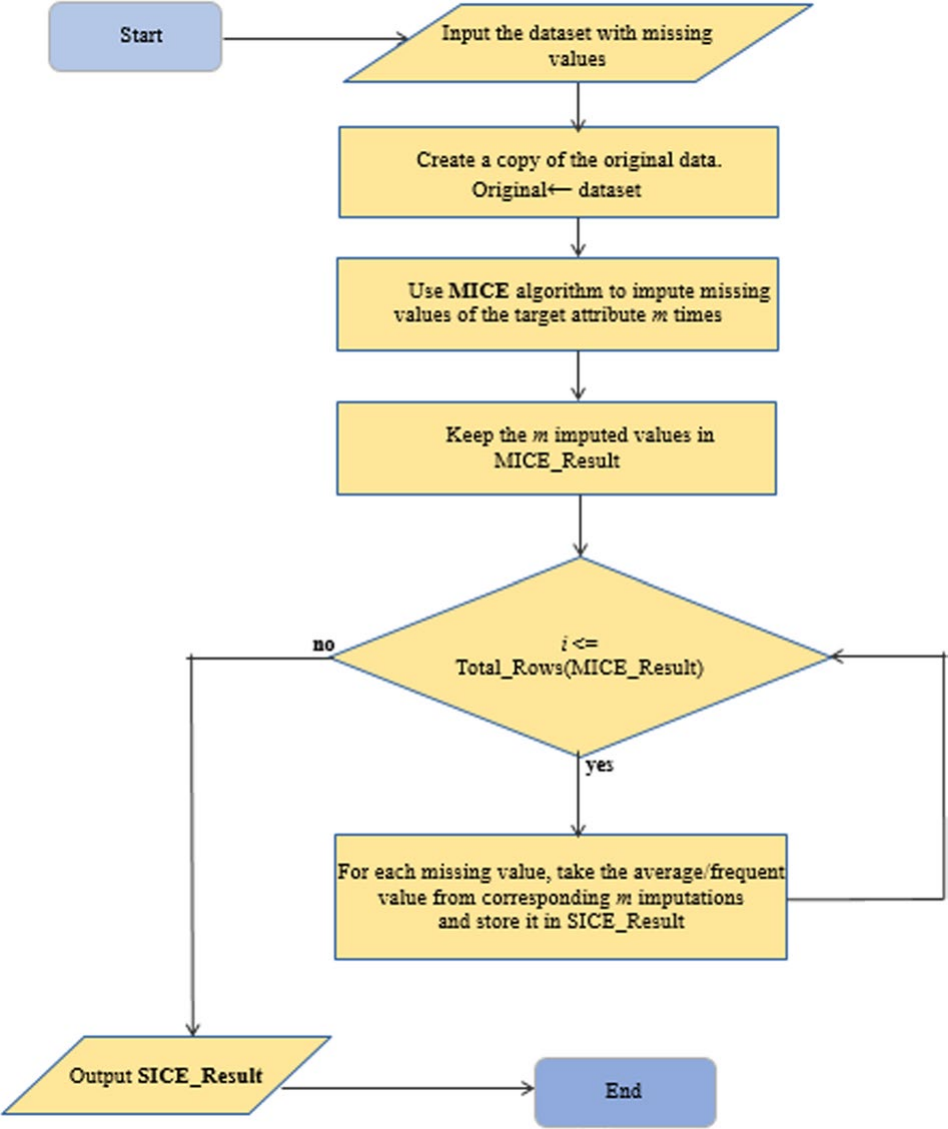
Flowchart of SICE

Multiple imputation based approach such as MICE is a better strategy for handling missing data than single imputation as multiple imputations consider the uncertainty of missing data. Multiple imputation strategy generates *m* values for a single missing data (where *m* is a user-defined number, usually set to 3 to 10). It is complex to use MICE in practical cases with a massive dataset as the data analyst has to preserve and analyze multiple datasets instead of one. In this section, we propose an algorithm **S**ingle Center **I**mputation from Multiple **C**hained **E**quation(*SICE*). It is an extension of the existing MICE algorithm. We have proposed two variants of SICE, namely SICE-Categorical and SICE-Numeric. Following Algorithm 1: SICE-Categorical imputes missing values of categorical attributes such as binary or ordinal attributes. For better understanding, we also present a flowchart of the SICE, which is applicable for both categorical and numeric versions in Fig. 3. It executes the MICE algorithm for user-defined *m* times and adds the results in an array. Then a missing value is replaced with the most frequent item of the array.

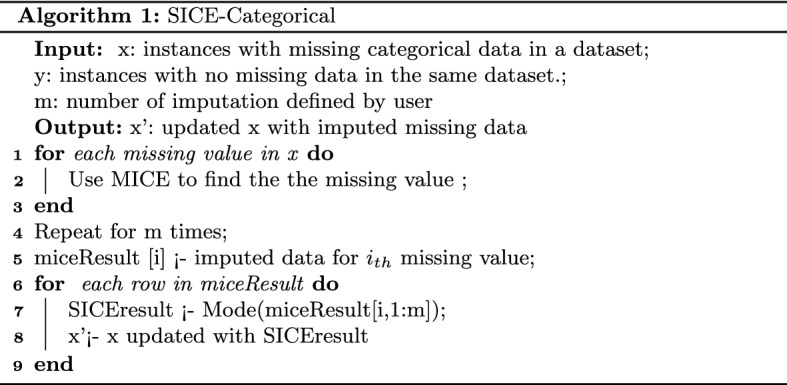



The Algorithm 2: SICE-Numeric imputes missing values for numeric attributes. It executes MICE algorithm for a user defined m times and adds the results of each iteration in an array. Then each missing value is replaced by the mean of its corresponding imputed value from the array.

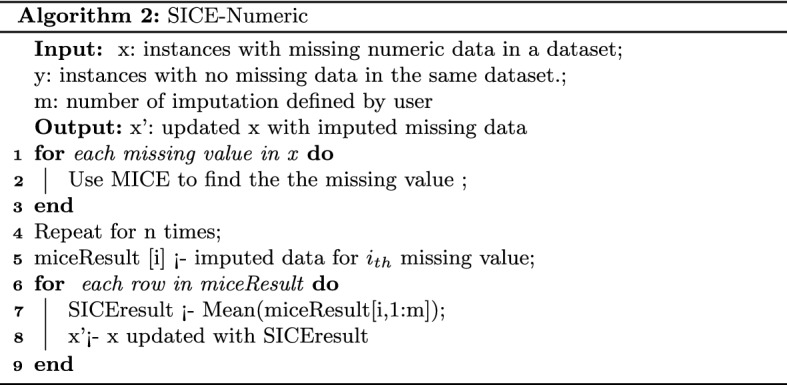



## Experimental design

**Fig. 4:**
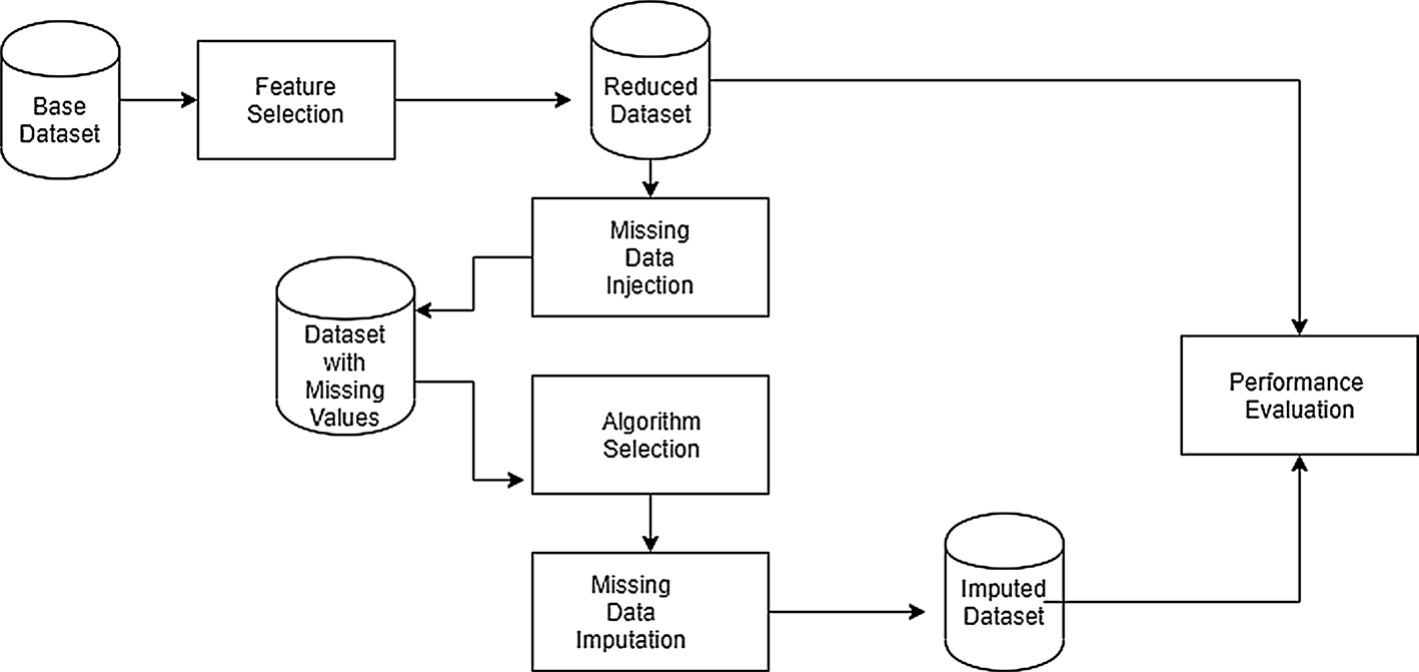
Block diagram of the system

The block diagram of our imputation and evaluation system is presented in Fig. 4. At first, a dataset with no missing values is selected as the base dataset. Then, feature selection is performed, depending on the base dataset, to remove unnecessary attributes. We name this as “Reduced Dataset,” which will be used later for performance evaluation of the imputation algorithms. Then we randomly inject 10% missing values to the target attribute of the backup copy of the reduced dataset. After that, we select different imputation algorithms based on the type of the target attribute, i.e., binary or numeric. Then we replace the missing values of the dataset using the selected algorithm of the previous step. In the final step, we evaluate the performance of different imputation algorithms using commonly used matrices such as accuracy, F-measure, or root mean square error.

The algorithms of the MICE package are available in the R environment [24]. The experiments were performed in R-studio. The implemented algorithms are selected based on the attribute type, such as numeric or binary, because some algorithms can able to impute selected attributes. In each experiment, missing values were injected using the ‘ampute’ function of the MICE package. Later, seven imputations were created using the “mice” function for each of the missing values. The researchers have claimed that to reach a satisfactory efficiency, three to ten number of imputations are sufficient [32]. After trying for different numbers of imputation, we empirically found seven to be a better performer. So we set the value of *m* (the number of iteration in MICE) to seven. We have run different algorithms by selecting the appropriate method parameter of the MICE function.

### Description of the datasets

We have used four datasets for our experiments. We have collected a local health dataset along with three public datasets from UCI Machine Learning Repository, the Dept. of Mathematics of ETH Zurich, and the Kaggle website. We will briefly describe the datasets here.

#### Local health dataset

We have collected patient records from a renowned healthcare center with proper ethical permission under the MoU with the Dept. of CSE, BUET. The dataset has 65 thousand health records, with 13 attributes containing demographic information and diagnosis data of patients. The attributes of the dataset fall into different categories, such as binary, ordinal, and numeric data types. At first, we performed feature elimination to get rid of unnecessary features or attributes, e.g., invoice number, etc. Then we performed the Chi-Square test on these attributes to discover the goodness of fit between them. After that, four attributes were found as significant. Among them, one was binary, two were nominal, and one was a numeric data type. For our experiments, we have injected 10% missing values in the dataset as per the guidelines of [33].

#### Hair eye color dataset

The second dataset that we have used is a publicly accessible dataset named HairEyeColor [34], which is a distribution of hair and eye color and gender in 592 statistics students. This dataset can be downloaded from the website of the Dept. of Mathematics of ETH Zurich. It is available in R-studio and can be accessed without the use of any external library. We have used the dataset to compare the performance of the binary imputation algorithms. Further details are placed in "Performance comparison for binary attributes" section.

#### UCI car dataset

We have collected another public dataset to test the performance of the algorithms for imputing ordinal values from the UCI Machine Learning Repository of the University of California, Irvine. The dataset is available at [35]. The number of rows in the dataset is 1728, and the number of columns is 7. Basic statistical descriptions of the target attribute and the results using this dataset are described in "Performance comparison for ordinal attribute" section.

#### Kaggle house price dataset

We have taken the last dataset from Kaggle, a popular online community of data scientists and machine learning practitioners. The dataset can be downloaded from [36]. It has 21614 rows and ten columns. Additional information regarding the target attribute and results is placed in "Performance comparison for numeric attribute" section.

## Results

We have implemented twelve algorithms to compare the performance of SICE. Among them, eight algorithms are included in the MICE package by default, three algorithms are available in different packages of R, and one algorithm (FURIA) is implemented using Weka. The list of the implemented algorithms for different attributes is presented in Table 6. Since each multiple imputation algorithm created seven predictions for each missing value, each algorithm provided seven different datasets as output. However, the result, we have mentioned for each multiple imputation based algorithm, are the best ones from its seven imputations. To evaluate the prediction quality in binary and ordinal attributes, we used Balanced Accuracy, Precision, Sensitivity, Specificity, and F-measure [37]. These properties were calculated and compared using the ‘confusionmatrix’ method from the ‘caret’ [38] package in R. For evaluating the performance of the algorithms on numeric attributes, We used root mean square error (RMSE) which is explained further in "Performance comparison for numeric attribute" section.

**Table 6:** List of existing algorithms implemented for comparison

Attribute Type		
Binary	Ordinal	Numeric
Implemented algorithms		
Logistic regression	Polytomous logistic regression (POLYREG)	Amelia
Predictive mean matching (PMM)	Predictive mean matching (PMM)	k nearest neighbors (kNN)
Fuzzy unordered rule induction algorithms (FURIA)	Linear discriminant analysis (LDA)	Predictive mean matching (PMM)
Support vector machine (SVM)	Classfication and regression tree (CART)	Bayesian linear regression (BLR)

### Performance comparison for binary attributes

Binary attributes are the attributes with two states only. An example of a binary attribute is *gender* when it has only two states: “Male” or “Female.” For binary attribute imputation, we have implemented predictive mean matching (PMM), logistic regression (LOGREG), Support Vector Machine (SVM), and Fuzzy Unordered Rule Induction Algorithm (FURIA).

We targeted the ‘Gender’ attribute of our local health dataset for imputation as it was the only binary attribute of the dataset. The attribute has 30549 female records and 34451 male records. 10% of total data were injected with missing values as per the guidelines of [33]. Logistic Regression and Predictive Mean Matching were implemented in R-studio using the “MICE’ package and FURIA was implemented in WEKA. Later, to verify SICE’s performance on binary attributes, we tested MICE and SICE on another publicly accessible dataset named HairEyeColor. More information about the dataset is presented in "Hair eye color dataset" section . We converted the “Age” attribute of our local health dataset later to binary attribute by using the following rule: Age < 18 “Minor”, Age ≥ 18 “Adult”. So total tested datasets and the target attributes for imputations are presented in Table 7.

**Table 7:** Datasets used for imputation of binary attribute

Dataset name	Targeted attribute name
HairEyeColor	Gender
Local health dataset	Gender
Local health dataset	Age (Binary)

**Fig. 5:**
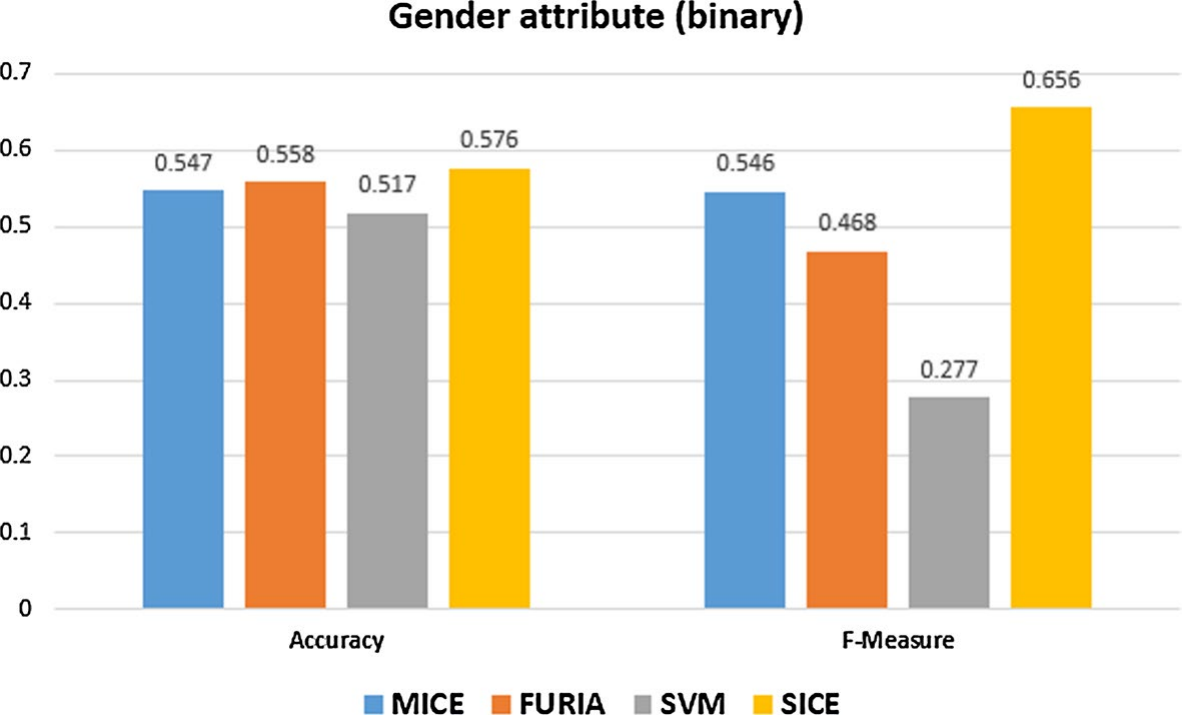
Accuracy and F-measure for four algorithms to impute gender attribute

We implemented MICE and SICE-Categorical using different methods such as PMM, LOGREG, etc. and found that for the binary attribute, SICE-Categorical performs better using the PMM method. The results are presented in Table 8. We can see that accuracy and F-measure of SICE is better than MICE, FURIA, and SVM. From Table 8, we can see that the F-measure of SICE is 0.656, whereas its closest competitor MICE’s F-measure, is 0.546. An illustration of the accuracy and F-measure of the algorithms are presented in Fig. 5.

**Fig. 6:**
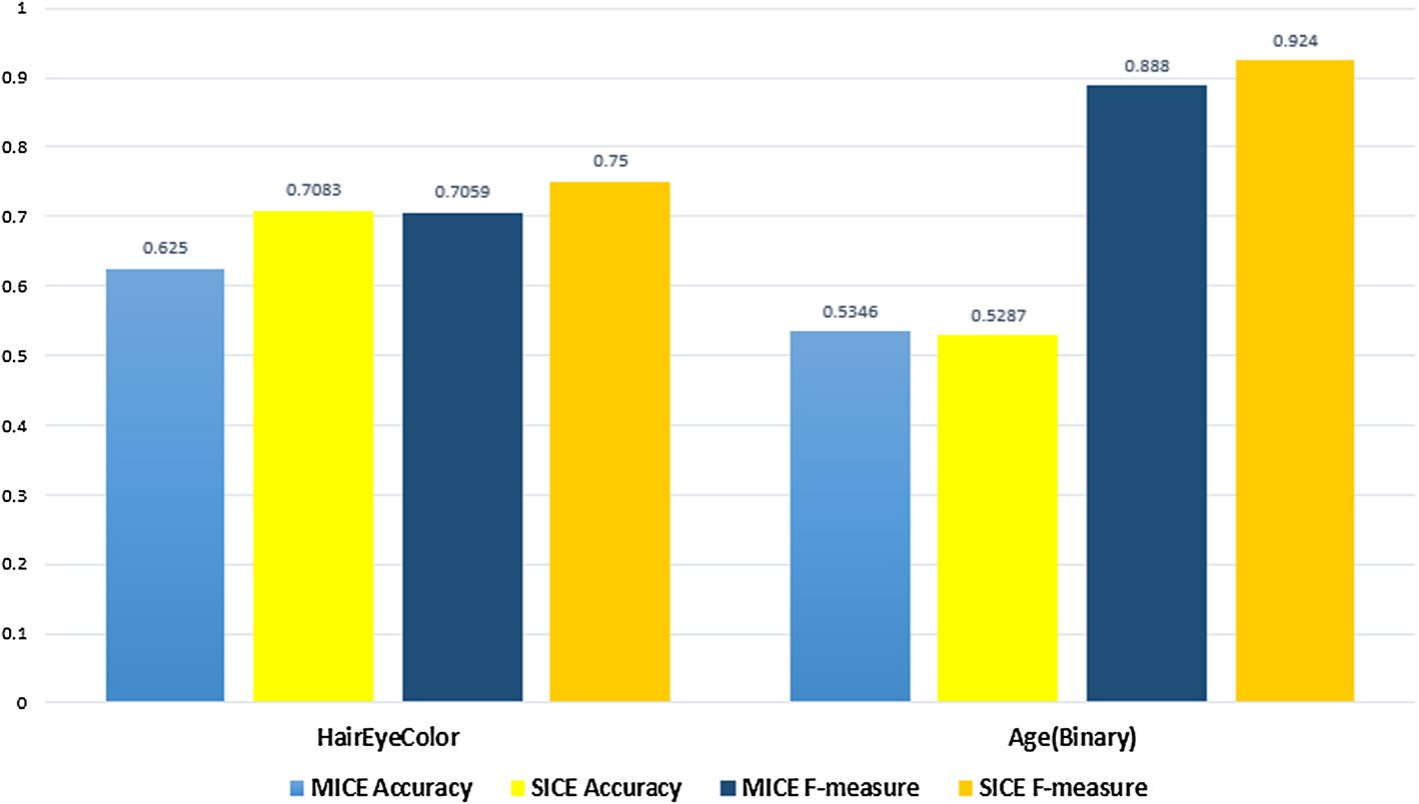
Performance comparison of MICE and SICE for additional binary datasets

The comparison of SICE with MICE for other datasets is shown in Fig. 6.

**Table 8:** Results for binary dataset “gender”

Algorithm	Accuracy	Sensitivity	Precision	Specificity	F-measure
MICE (PMM)	0.546	0.546	0.546	0.547	0.546
FURIA	0.558	0.558	0.597	0.128	0.468
SVM	0.517	0.188	0.522	0.847	0.276
SICE (PMM)	0.576	0.656	0.656	0.499	0.656

### Performance comparison for ordinal attribute

Ordinal attributes are categorical attributes that have specific levels and maintain order among the levels. For example, if age is converted to categorical data, then that is an ordinal attribute because it has some specific levels with orders, namely- Infant, Child, Adolescent, Adult, and Senior. We have used the MICE and RKEEL packages in R for our experiments. We have imputed the Age attribute of our local health dataset described in "Local health dataset" section . The variable was initially in a specific date format. First, we converted it into a numeric attribute. We further changed it into ordinal attribute “AgeLevel” by categorizing it into ‘Children,’ ‘Young,’ ‘Adult,’ and ‘Senior,’ by following the guidelines of [39]. Around 10% of missing data was injected in the target attribute “AgeLevel.” Number of rows: 64999, Number of columns: 04, Data in the target (“AgeLevel”) column: children–4082, Young–6584, Adult–44469, Senior–9864

**Fig. 7:**
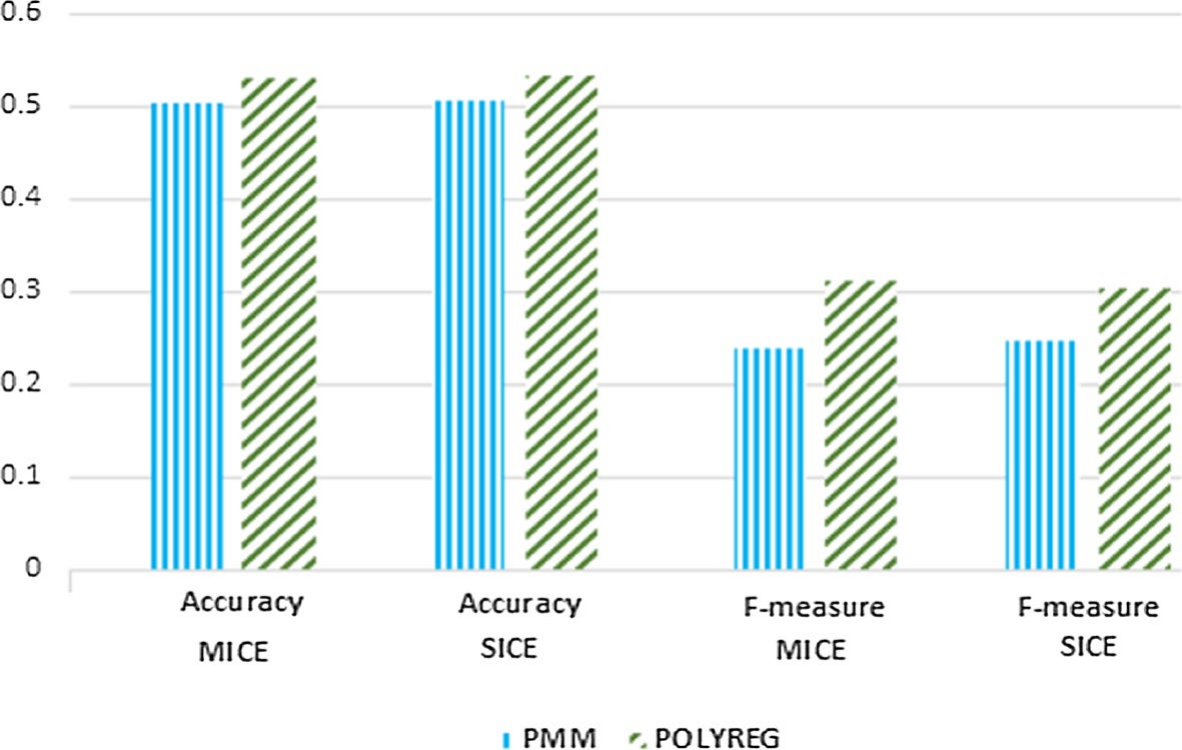
Performance of MICE and SICE for ordinal data using PMM and POLYREG

For the imputation of missing ordinal data, we implemented four algorithms, PMM, POLYREG, CART, and LDA. The results obtained using MICE and SICE-Categorical are tabulated in Table 9. We can see that performance of both MICE and SICE is similar. Figure 7 depicted the performance of MICE and SICE-Categorical using PMM and POLYREG, methods for imputing ordinal missing data. Both MICE and SICE have shown similar performance with no convincing results. As for ordinal or nominal attributes, there are many choices for a single value; it is difficult to predict the value correctly. However, for a large dataset, the result is expected to improve.

**Table 9:** Performance of MICE and SICE for ordinal attribute using local health dataset

Algorithm	MICE		SICE	
	Accuracy	F-measure	Accuracy	F-measure
PMM	0.503	0.246	0.505	0.238
POLYREG	0.531	0.303	0.532	0.312
CART	0.537	0.318	0.536	0.283
LDA	0.562	0.353	0.561	0.341

**Fig. 8:**
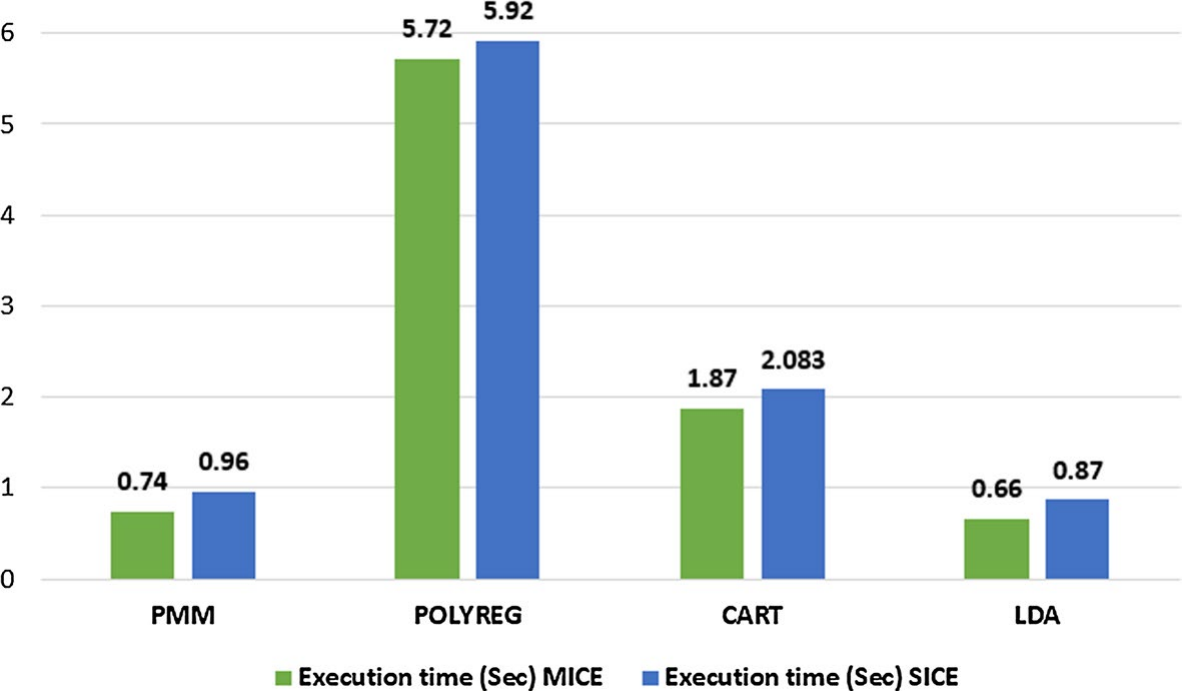
Comparison of execution time of MICE and SICE to impute UCI car dataset

We have also collected a public dataset to impute ordinal values from the UCI Machine Learning Repository. Details are presented in Section 4.1.3. Some basic statistical descriptions of the target attribute are given below. Number of rows: 1728, Number of columns: 7, Data in the target (“Target”) column: ‘acc’–384, ‘good’–69, ‘unacc’–1210, ‘vgood’– 65. The accuracy of MICE and SICE using four methods: PMM, POLYREG, CART, and LDA, are presented in Table 10. We can see from the results that our proposed SICE scored the highest accuracy (93.06) and F-measure (81.83) using the CART method as a parameter. The execution time of MICE and SICE in seconds are presented in Fig. 8. We can see that MICE using the LDA method has the lowest execution time (0.66 seconds), and SICE has slightly higher execution time (0.87 seconds).

**Table 10:** Performance of MICE and SICE for ordinal attribute using UCI car dataset

Algorithm	Accuracy		F-measure	
	MICE	SICE	MICE	SICE
PMM	62.42	74.56	23.41	29.51
POLYREG	83.81	89.59	72.35	76.29
CART	89.01	93.06	76.88	81.83
LDA	80.92	80.92	60.63	64.92

### Performance comparison for numeric attribute

Numerical attributes are attributes with Numbers. These attributes can be either integer or decimals. An example of the numeric attribute can be the weight of people in kilograms or pounds. We have performed the imputation of a numeric attribute using four different algorithms. They are kNN, Amelia, PMM, and BLR. PMM and BLR algorithms are included in the MICE package of R. Amelia algorithm has its own package in R named “amelia.” The kNN algorithm is available under the “class” package of R. The experiment was conducted targeting the numeric attribute ‘Age’ in our local health dataset. Age of the people was in years, and other attributes that were present during imputation are one binary attribute and two nominal attributes.

Here the target numeric attribute is “age.” We have randomly injected 10% missing value (6500 value missing). Some useful statistics of the values are given below. Min = 1, Max = 100, Range = 1 to 100, Mean = 43.85, Median = 45, Standard Deviation = 18.79, Skewness = − 0.08189913, kurtosis = − 0.5296097.

The results obtained are tabulated in Table 11. To evaluate the algorithms, we have calculated and compared the Root Mean Squared Error (RMSE) of each algorithm. The RMSE calculates the absolute fit of the model, and therefore it depicts how closely predicted values are related to the real values. The lower the RMSE (error value), the better the prediction of an algorithm. To calculate RMSE, we used the following formula:

$$\begin{aligned} \hbox {RMSE}=\sqrt{\frac{1}{n}\varSigma _{i=1}^{n}{({real\_value_i -predicted\_value_i})^2}} \end{aligned}$$

It can be seen that our proposed SICE-Numeric using Bayesian Linear Regression (BLR) method as a parameter gives better results than other investigated algorithms. The prediction error of our proposed SICE is 19.47, which is the lowest compared to its competitors. MICE algorithm using the Predictive Mean Matching (PMM) method achieved the second-lowest error, which is 21.85. On the other hand, the Amelia algorithm has the lowest execution time, which is 12 seconds.

**Table 11:** Performance of the algorithms for numeric attribute of local health dataset

Algorithm	RMSE score	Execution time (s)
SICE (BLR)	*19.47*	19
MICE (PMM)	21.85	282
MICE (BLR)	24.47	18
Amelia	25.6	*12*
kNN	25.25	154

We have taken the second dataset for numeric imputation from Kaggle, Details of the dataset is presented in "Kaggle house price dataset" section. The target numeric attribute is “price”. We have randomly injected 10% missing value (2161 value missing). We converted the price column unit from $ to k$. Some useful statistics of the values are given below. Min = 75, Max = 7700, Range = 75 to 7700, Mean = 540.18, Median = 450, Standard Deviation = 367.36, Skewness = 4.021157, kurtosis = 34.51071.

**Fig. 9:**
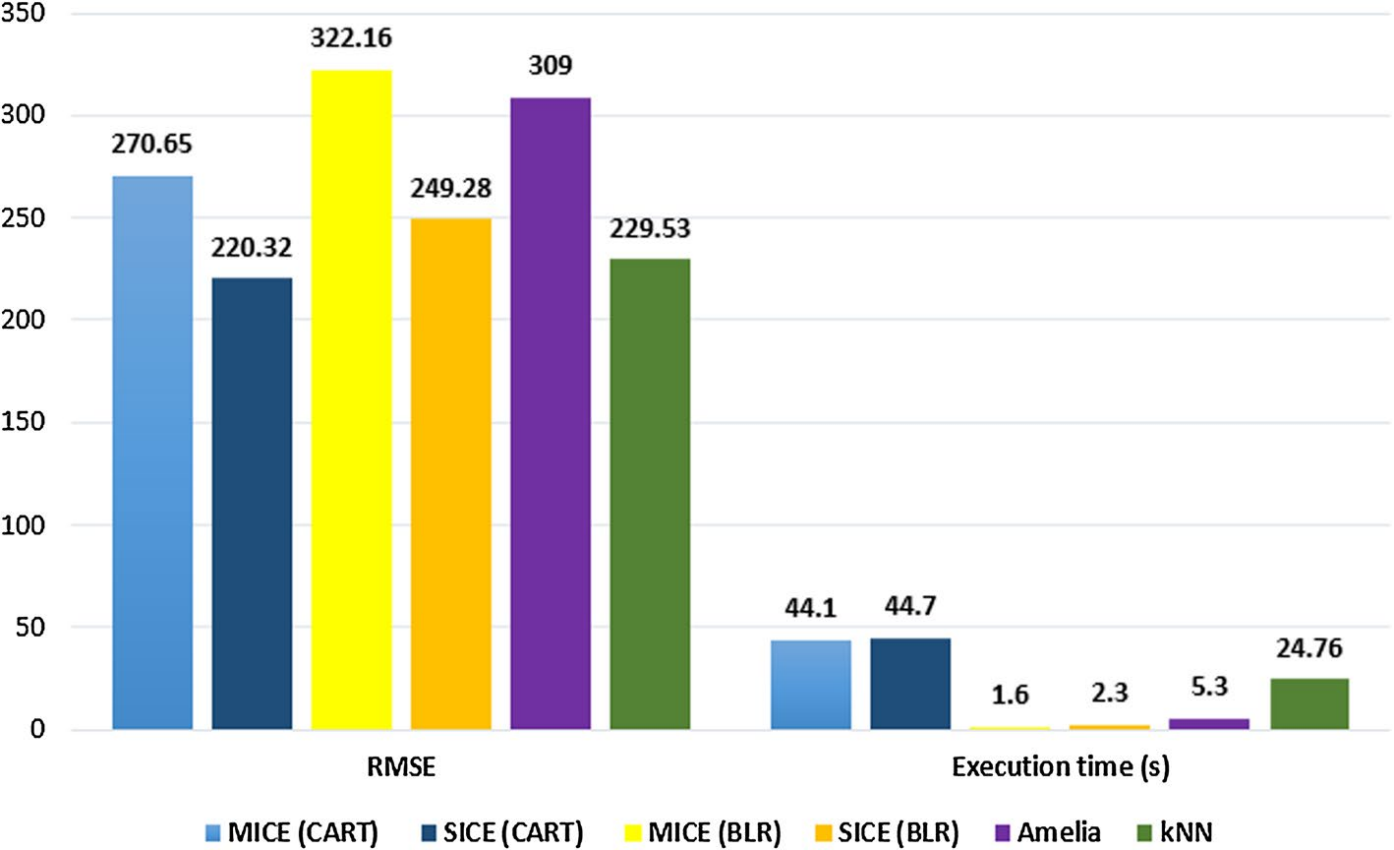
Performance of algorithms to predict house prices

We have run MICE and SICE to impute the dataset using CART and BLR methods. We have also run Amelia and kNN algorithms to impute missing values. Price prediction error and execution time are presented in Fig. 9. We can see that our proposed algorithm SICE using the CART method imputes the dataset with the lowest RMSE error 220, where its close competitor is kNN with RMSE 229. On the other hand, MICE (BLR) has the lowest execution time 1.6 second, and its close competitor is SICE (BLR) with 2.3 seconds.

## Discussions and limitation

In this paper, we have proposed an algorithm **S**ingle Center **I**mputation from Multiple **C**hained **E**quation (SICE) with two variants SICE-Categorical and SICE-Numeric to impute missing categorical and numeric data. From the "Results" section, it can be observed that SICE-Categorical shows better performance over MICE and other implemented algorithms for imputing missing binary data. For all three datasets, SICE achieved 10% to 20% accuracy and F-measure than its competitors. SICE-Numeric also performs better predictions with less RMS error for imputing missing numeric data. That means it provides closer prediction to the correct value than its competitors.

One limitation of SICE-Categorical is that it could not show better performance than MICE for the case of ordinal data. One of the main challenges here is that, for the case of ordinal or nominal data, there may be many states. For example, there are many options for a missing blood group of a person, e.g., B+, B−, O+, O−, AB+, etc. So, it is difficult to impute missing nominal data correctly. In the future, we will focus on improving the SICE-Categorical so that it can perform better for imputing ordinal and nominal data. Another point to notice is that our proposed technique SICE requires slightly higher execution time than MICE (See Fig. 9). This is logical as we have extended MICE by adding some additional steps in it. This little increase in the execution time can be overlooked as missing data imputation step is performed *offline* in the preprocessing step of a data analytics project.

## Conclusion

The significance of the imputation of missing data is very high in data analytics. Finding a suitable method of missing data imputation for all type of dataset is very challenging. Single imputation based missing data handling methods are easy to implement but may provide biased imputations, according to statisticians. On the other hand, multiple imputation based methods consider the uncertainty of a dataset and generate a set of plausible values for each missing data, which are complex to implement. MICE package in R provides the platform to implement Multivariate Imputation of Chained Equations (MICE) technique and support twenty-two methods. In this paper, we have proposed an algorithm *SICE* for missing data imputation. It is an extension of the popular MICE algorithm. We have presented two variants of SICE: SICE-Categorical and SICE-Numeric to impute binary, ordinal, and numeric data. We have implemented twelve existing imputation methods and compare their performance with SICE. Experimental results with four different datasets show that our proposed method SICE performed better for the imputation of binary and numeric data. In terms of F-measure, the improvement is around 20%, and in terms of error reduction, the improvement is around 11%. The execution time of SICE is almost equal to MICE. So, we can say that SICE is an excellent choice for missing data imputation, especially for massive datasets where MICE is impractical to use because of its complexity. In the future, we will extend the SICE algorithm for improving its performance further, especially for nominal data.

## Data Availability

The availability of all data sources is described in the article with reference.

## Abbreviations

MICE: Multivariate Imputation by Chained Equation
SICE: Single Center Imputation from Multiple Chained Equation
UCI: University of California, Irvine
ETH Zurich: Eidgenössische Technische Hochschule Zurich
MCAR: Missing Completely at Random MAR Missing at Random
MNAR: Missing Not at Random
NA: Not Available FURIA Fuzzy Unordered Rule Induction Algorithm
kNN: k-Nearest Neighbor
SVM: Support Vector Machine
RMSE: Root Mean Square Error
bPCA: Bayesian Principal Component Analysis
FKM: Fuzzy K-Means
FRNN: Fuzzy-Rough Nearest Neighbor
FRNNI: Fuzzy-Rough Nearest Neighbors Imputation
VQRS: Vaguely Quantified Rough Sets
OWABRS: Ordered Weighted Average Based Rough Sets
BLR: Bayesian Linear Regression
PMM: Predictive Mean Matching
LOGREG: Logistic Regression
POLYREG: Polytomous Logistic Regression
LDA: Linear Discriminant Analysis
CART: Classification and Regression Tree
BUET: Bangladesh University of Engineering and Technology
